# Human Defensin 5 Inhibits *Plasmodium yoelii* Development in *Anopheles stephensi* by Promoting Innate Immune Response

**DOI:** 10.3390/tropicalmed9080169

**Published:** 2024-07-25

**Authors:** Tingting Liu, Jing Wang, Xin Li, Shasha Yu, Dan Zheng, Zhilong Liu, Xuesen Yang, Ying Wang

**Affiliations:** 1 Department of Tropical Medicine, College of Military Preventive Medicine, Army Medical University, Chongqing 400038, China; liutingting@tmmu.edu.cn (T.L.);; 2 School of Public Health, the Key Laboratory of Environmental Pollution Monitoring and Disease Control, Ministry of Education, Guizhou Medical University, Guiyang 550025, China

**Keywords:** human defensin 5, *Anopheles stephensi*, *Plasmodium yoelii*, Toll signaling pathway

## Abstract

Malaria poses a serious threat to human health. Existing vector-based interventions have shortcomings, such as environmental pollution, strong resistance to chemical insecticides, and the slow effects of biological insecticides. Therefore, the need to develop novel strategies for controlling malaria, such as reducing mosquito vector competence, is escalating. Human defensin 5 (HD5) has broad-spectrum antimicrobial activity. To determine its effect on *Plasmodium* development in mosquitoes, HD5 was injected into *Anopheles stephensi* at various time points. The infection density of *Plasmodium yoelii* in *An. stephensi* was substantially reduced by HD5 treatment administered 24 h prior to infection or 6, 12, or 24 h post-infection (hpi). We found that HD5 treatment upregulated the expression of the innate immune effectors TEP1, MyD88, and Rel1 at 24 and 72 hpi. Furthermore, the RNA interference of MyD88, a key upstream molecule in the Toll signaling pathway, decreased the HD5-induced resistance of mosquitoes against *Plasmodium* infection. These results suggest that HD5 microinjection inhibits the development of malaria parasites in *An. stephensi* by activating the Toll signaling pathway.

## 1. Introduction

Malaria, transmitted by the bite of female *Anopheles* mosquitoes, is a parasitic disease that caused an estimated 249 million malaria cases and 608,000 deaths in 2022 [1]. Vector-based interventions are the principal currently available methods for reducing malaria. However, the long and widespread use of chemical insecticides not only causes environmental pollution but also promotes resistance in mosquitoes. Biological insecticides show relatively slow effects and short killing efficiencies, in addition to the problem of resistance [2]. Therefore, there is an urgent need to identify novel strategies for combating malaria and reducing mosquito vector competence [3]. In addition to the extermination of mosquitoes, malaria transmission can be blocked by interfering with the development of *Plasmodium*, thereby reducing the vector capacity of *Anopheles* mosquitoes.

A female mosquito ingests a blood meal infected with *Plasmodium* gametocytes. The fertilization of the gametes results in the formation of zygotes in the midgut. Then, they develop into motile ookinetes and invade the midgut epithelium 18–26 h post-infection [4]. As ookinetes migrate through the midgut epithelium and oocysts develop, mosquitoes defend against *Plasmodium* by the Toll and Imd NF-κB immune signaling pathways, which contain some key immune molecules, such as TEP1, MyD88, Rel1, and Rel2 [5]. Insect antimicrobial peptides (AMPs) defend against the infection of microorganisms by activating different signaling pathways, such as Toll-like receptors (TLRs), NF-κB, MAPK, and Janus kinase–signal transducer and activators of transcription (JAK-STAT) pathways [6]. Defensins are cys-rich AMPs with three pairs of intramolecular disulfide bonds [7]. They are an important component of innate immunity in mammals, insects, and plants. Defensin is involved in various biological reactions, including host cellular immunity responses against a variety of pathogenic microorganisms [8]. Defensins reportedly promote the innate and adaptive immune regulation of hosts [9]. Insect defensins are synthesized mainly in the fat body and then spread by the hemolymph throughout the entire body to fight infection [10]. The injection of two insect defensins, *Aeschna cyanea* (dragon fly) and *Phormia terranovae* (flesh fly), exhibited a profound toxic effect on the oocysts of *Plasmodium gallinaceum* in *Aedes aegypti* and on isolated sporozoites [11]. Although mosquito defensins are required for the antimicrobial defense against Gram-positive bacteria, they have no significant effect on the development of the parasite midgut stages of *Plasmodium berghei* in *Anopheles gambiae* [12].

Mammalian defensins have more broad-spectrum and highly effective antimicrobial activity. Between insects and mammals, defensins are differentiated by a conserved cysteine motif [13]. Human defensin 5 (HD5) is a member of the alpha family of short antimicrobial peptides secreted by Paneth cells located at the base of intestinal crypts [14,15]. Research showed that HD5 displays a parasiticidal role against *Toxoplasma gondii* [16]. Human primary endocervical epithelial cells (HPECs) participate in mucosal immune defense by upregulating the secretion of HD5 by activating Toll-like receptor 4 (TLR4) [17].

*Anopheles stephensi* is an endemic vector of malaria in Southeast Asia [18]. The animal models of *An. stephensi* and *Plasmodium yoelii*, which infect rodents, are often used to study the relationship between the vector and disease [19]. Hence, we investigated whether HD5 can regulate innate immunity in *An. stephensi* and affect the development of *P. yoelii*. In the present study, we showed that HD5 effectively inhibited *P. yoelii* infection in *An. stephensi* by activating the Toll signaling pathway. This is the first study to decipher the effect of exogenous HD5 on *Plasmodium* development in mosquitoes and its innate immunity-related mechanisms. It may direct us to understand the underlying antimalarial mechanisms of exogenous defensins into mosquitoes to investigate the possibility of genetically manipulating the insect’s immune system.

## 2. Materials and Methods

### 2.1. Mosquito Rearing and Infection

The *An. stephensi* Hor strain was maintained at 28 °C and 70–80% relative humidity with a 12 h light/dark photocycle, according to the standard rearing procedures in the laboratory. The red fluorescent protein transgenic *P. yoelii* BY265 strain was kindly donated by Professor Wenyue Xu from the Army Medical University and passed through the mice. Female *An. stephensi* mosquitoes (3–5 days old) were allowed to feed on *P. yoelii*-infected 4–6-week-old Kunming mice with 5–10% parasitemia at 24 °C, as described previously [20]. The mosquitoes were anesthetized with CO_2_ and dissected at day 9 post-infection. *Plasmodium* oocysts were counted under a fluorescence microscope. The infection rate and density were determined.

### 2.2. HD5 Treatment

The HD5 polypeptide was directly synthesized using chemical methods with 95% purity and was kindly donated by Professor Cheng Wang from the Institute of Combined Injury of the Army Medical University. The dry powder of HD5 was dissolved into the stock solution (1 mg/mL) and then diluted with ddH_2_O to a final working concentration (200 μg/mL) before injection. Female *An. stephensi* (3–5 days old) were intrathoracically injected with 69 nL HD5. Age-matched mosquitoes injected with 69 nL ddH_2_O served as controls. The injection was conducted 24 h prior to infection with *P. yoelii* or 6, 12, 24, or 72 h post-infection (hpi).

### 2.3. RNA Isolation, cDNA Synthesis, and Quantitative PCR

RNA expression was tested using pooled RNA from 10 mosquitoes. Ten female *An. stephensi* mosquitoes from each group were anesthetized with CO_2_ and used for gene transcript analysis 24 and 72 h post-infection (hpi). Total RNA was extracted in accordance with the manufacturer’s instructions for the HiPure Universal RNA Mini Kit (Magen, Guangzhou, China) and reverse-transcribed into cDNA using the Reverse Transcription Kit (Takara, Dalian, China). Thereafter, real-time quantitative PCR was performed using the KAPA SYBR^®^ FAST qPCR Kit (KAPA Biosystems, Wilmington, MA, USA) with a Bio-Rad CFX96 Touch™ real-time PCR instrument (Bio-Rad, Hercules, CA, USA) to determine the transcriptional levels of immune molecules, such as thioester-containing protein 1 (TEP1), MyD88, Rel1, Imd, Rel2, and Caspar, using the conserved *S7* as the internal reference gene [21]. The primers used are listed in Table 1. The expression of each gene relative to the ribosomal S7 RNA was determined using the 2^−ΔΔCT^ method [22].

### 2.4. Western Blot

Fifteen *An. stephensi* mosquitoes from each group were homogenized in RIPA lysis buffer (50 mM Tris, pH 7.4; 150 mM NaCl; 1% sodium deoxycholate; 1% Triton X-100; 1 mM EDTA; 0.1% SDS; 1× protease inhibitor; and 1× phosphatase inhibitor) 24 and 72 h after blood meal. Total protein was extracted, denatured at 95 °C for 5 min. Then, protein samples were separated using 10% sodium dodecyl sulfate–polyacrylamide gel electrophoresis and transferred onto a PVDF membrane for 1 h at 100 V. The membrane was blocked in 5% skimmed milk for 1 h at room temperature, then incubated overnight with a 1:2000 TEP1 antibody (designed by our laboratory, synthesized by Genecreate, Wuhan, China) and reference protein β-actin antibody at 4 °C. The membranes were washed three times with 1× TBST for 5 min and then incubated with an anti-rabbit secondary antibody for 1 h at room temperature. After washing as described above, membrane signals were acquired using the Chemi DOCTM MP Imaging System (Bio-Rad).

### 2.5. RNA Interference

RNA interference was conducted to further confirm the role of the Toll signaling pathway in the effect of HD5 treatment on the susceptibility of *An. stephensi* to *P. yoelii*. *An. stephensi* cDNA was subjected to PCR using TEP1 and MyD88 gene-specific primers with a 5′ extension of T7 promoter tags (5′-TAATACGACTCACTATAGGG-3′) as described previously [23]. A GFP fragment was used to construct control double-stranded RNA (dsRNA) (Table 2). The dsRNA was transcribed in vitro using PCR products as templates with the MEGAscript T7 Kit (Ambion Life Technologies, Austin, TX, USA) following the manufacturer’s instructions. Female mosquitoes (3–5 days old) were intrathoracically injected with 69 nL dsTEP1 or dsMyD88 using a Nanoject II microinjector (Drummond Scientific Co., Bromall, PA, USA). Equivalent amounts of dsGFP were used as a control. Gene silencing efficiency was detected three days after dsRNA injection by qRT-PCR, as described above. The mosquitoes were challenged with *P. yoelii* BY265RFP infection via blood-feeding. *Plasmodium* oocysts in mosquitoes were microscopically counted eight days after infection. The infection rates and densities were determined.

### 2.6. Statistical Analysis

All statistical analyses were performed using GraphPad Prism (version 8.0) and SigmaStat (version 3.5) software. The Chi-square test was used to analyze the infection rate. Student’s *t*-test was used to compare normally distributed data with equal variance. The Mann–Whitney U-test was used for non-normally distributed data to compare oocyst counts between the HD5 and control groups. Statistical significance was set at *p* < 0.05. Bonferroni corrections were conducted for multiple comparisons.

## 3. Results

### 3.1. HD5 Injection Prior to Infection Reduced P. yoelii Infection Density in An. stephensi

**Figure 1:**
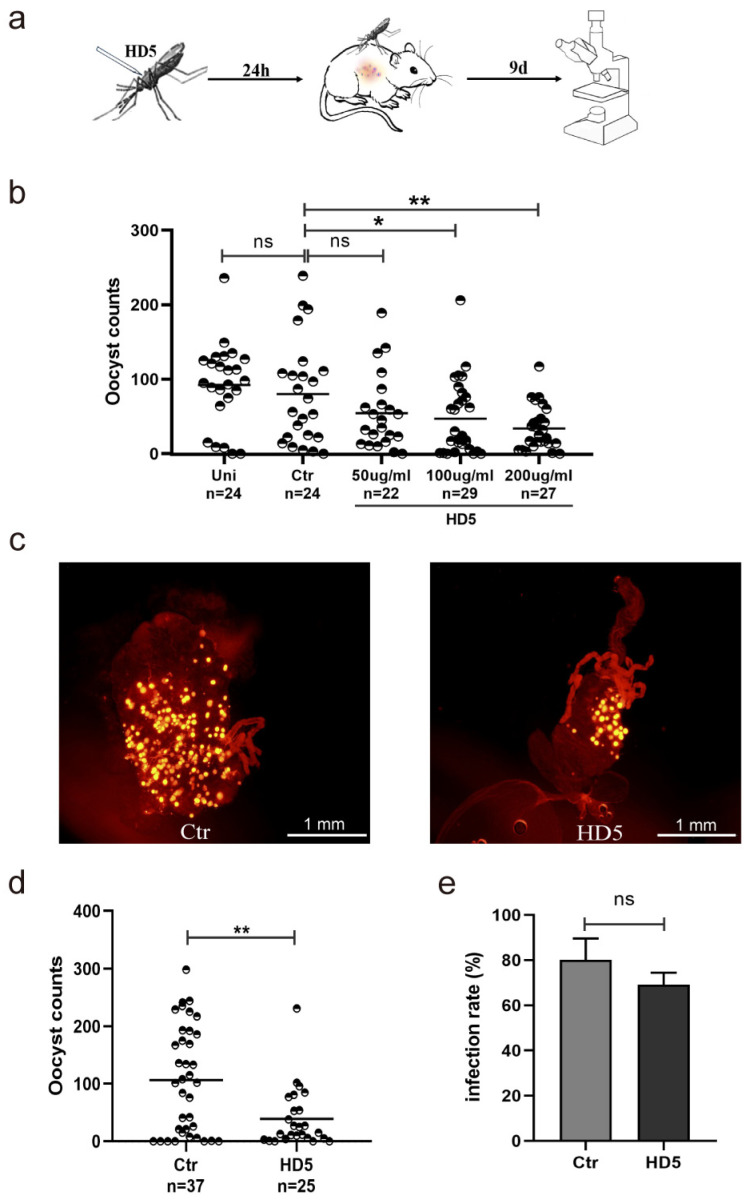
HD5 injection prior to infection decreased the density of oocysts in *An. stephensi*. (**a**) A schematic overview of HD5 treatment in *An. stephensi*. (**b**) The effects of different concentrations of HD5 on *Plasmodium* development in mosquitoes (**c**) The infection of *P. yoelii* in the midguts of *An. stephensi* (yellow dots represent oocysts). (**d**) The infection density of *Plasmodium* in mosquitoes between the HD5 (200 μg/mL) and control groups. (**e**) The infection rates of the HD5 (200 μg/mL) and control groups. Each dot represents the oocyst of an individual mosquito (**a**,**d**). Horizontal black bars indicate the median values. Significance was determined using Mann–Whitney tests in (**a**,**d**), and using the Chi-square test in (**e**). *, *p* < 0.05; **, *p* < 0.01; ns, no significant difference. Uni, un-injection; Ctr, control, injection with ddH_2_O; HD5, injection with HD5.

To explore whether HD5 injection pre-treatment affects the development of *Plasmodium* oocysts in *An. stephensi*, the mosquitoes were intrathoracically injected with HD5 24 h prior to infection. Oocysts from the control and HD5 treatment groups were counted at day 9 post-infection (Figure 1a). Infection rates and densities were compared between the two groups. And the effects of different HD5 concentrations on the parasite development in *An. stephensi* were explored. As a result, the low concentration of 50 μg/mL had minimal effect on parasite infection. The administration of HD5 at concentrations of 100 and 200 μg/mL led to substantially lower infection densities of *Plasmodium* than that in the control group (Figure 1b). To further confirm the inhibitory effect of 200 μg/mL HD5 on oocyst development, an independent experiment with this concentration was conducted. It was shown that 200 μg/mL HD5 treatment decreased oocyst count by 61% compared to the control group (Figure 1c,d). Thus, all the following experiments were conducted at 200 μg/mL. No significant difference in infection prevalence was noted between the control and HD5 treatment groups (Figure 1e). These data suggest that HD5 treatment before infection influenced oocyst development in *An. stephensi* and reduced the infection density.

### 3.2. HD5 Treatment Post-Infection Inhibited P. yoelii Infection in An. stephensi

**Figure 2:**
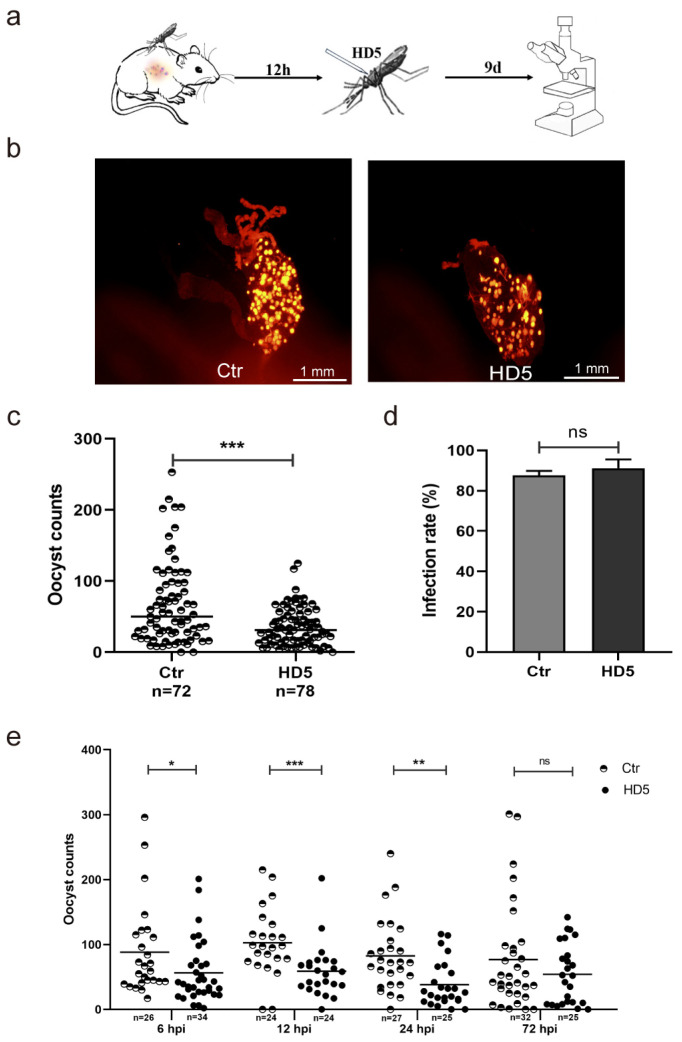
Post-infection HD5 injection inhibits *P. yoelii* infection in *An. stephensi*. (**a**) A schematic overview of HD5 microinjection into *An. stephensi*. (**b**) The infection of *P. yoelii* in the midguts of *An. stephensi* injected with ddH_2_O or HD5 at 12 hpi (yellow dots represent oocysts). (**c**) The infection density of *Plasmodium* in mosquitoes injected with ddH_2_O or HD5 at 12 hpi. (**d**) *Plasmodium* infection rates in *An. stephensi* injected with ddH_2_O or HD5 at 12 hpi. (**e**) The oocyst counts of *Plasmodium* in *An. stephensi* injected with ddH_2_O or HD5 at different time points. Each dot represents the oocyst of an individual mosquito. Horizontal black bars indicate the median values (**c**,**e**). Significance was determined using Mann–Whitney tests in (**c**,**e**) and using the Chi-square test in (**d**). *, *p* < 0.05; **, *p* < 0.01; ***, *p* < 0.001; ns, no significant difference. Ctr, control, injection with ddH_2_O; HD5, injection with HD5.

To explore whether injecting HD5 into mosquitoes after blood-feeding affects parasitic infection, HD5 was intrathoracically injected into *An. stephensi* post-infection. The mosquitoes were dissected, and the oocysts were counted at day 9 post-infection (Figure 2a). We first determined the effect of HD5 injection at 12 hpi on the development of *Plasmodium* in mosquitoes. The results showed that the HD5 injection substantially reduced oocyst count compared to the control group without affecting infection rates (Figure 2b–d). The inhibitory effect of HD5 on *Plasmodium* development in mosquitoes was observed when injection was conducted at 6, 12, 24, or 72 hpi. The results showed that the injection of HD5 led to a 32% decrease at 6 hpi, a 44% decrease at 12 hpi, a 44% decrease at 24 hpi, and a 23% decrease at 72 hpi compared to the control groups (Figure 2e). These results indicated that HD5 could intervene in the development of the early stage of the oocyst. In summary, HD5 effectively inhibited *P. yoelii* development in *An. stephensi* if HD5 was injected prior to infection or administered early after infection.

### 3.3. HD5 Treatment Upregulated TEP1 Expression in An. stephensi

**Figure 3:**
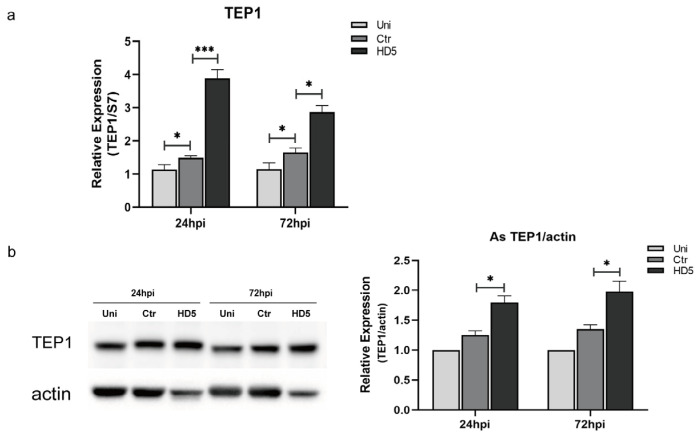
HD5 affects TEP1 expression in *An. stephensi* infected with *P. yoelii*. TEP1 transcriptional levels detected by real-time quantitative PCR (**a**) and protein levels determined by Western blot analysis (**b**). The bar chart represents the quantification of the signal intensities of TEP1 relative to actin from three independent replicates determined by ImageJ software 5.0 (**b**). The relative expression was set as 1 for the Uni group at each time point. Error bars indicate standard errors. Significance was determined using *t*-tests. *, *p* < 0.05; ***, *p* < 0.001. Uni, un-injection; Ctr, control, injection with ddH_2_O; HD5, injection with HD5.

Studies have indicated that TEP1 plays a crucial role in mosquito resistance against malaria parasites [24]. The midgut epithelium is invaded and crossed by *Plasmodium* ookinetes, triggering a robust TEP1-involved, complement-like immune response [25,26]. As the main protein responsible for mediating *Plasmodium* lysis, TEP1 binds to the surface of ookinetes and mediates *Plasmodium* lysis [27]. To investigate whether HD5 treatment affected the expression of TEP1, real-time quantitative PCR and Western blotting were performed to detect changes in the transcription and protein levels of TEP1 by HD5 injection at 12 hpi. The results showed that HD5 treatment upregulated both the transcriptional and protein levels of TEP1 in mosquitoes at 24 and 72 hpi, compared to those in the control groups (Figure 3a,b). Additionally, the damage of injection upregulated the transcription levels of TEP1 but had minimal effect on protein levels comparing the un-injected and injected with ddH_2_O groups. These results suggest that HD5 treatment could increase the expression of TEP1 and improve the immune response of mosquitoes against malaria parasites.

### 3.4. Toll Signaling Pathway Was Upregulated by Injection with HD5

**Figure 4:**
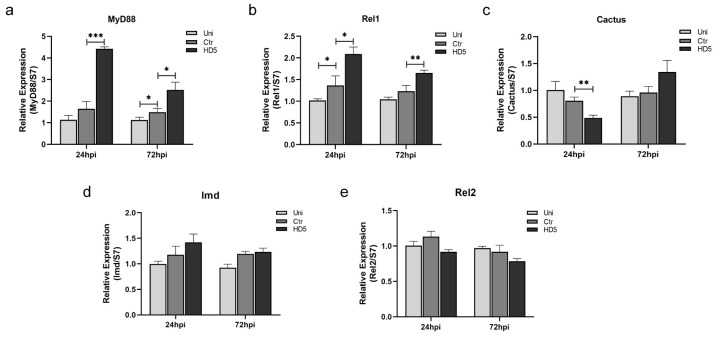
The impact of HD5 treatment on the activation of the Toll and Imd signaling pathways in *An. stephensi*. A gene expression analysis of MyD88 (**a**), Rel1 (**b**), Cactus (**c**), Imd (**d**), and Rel2 (**e**). The expression levels of targeted genes were normalized to S7. The relative expression was set as 1 for the Uni group at each time point. Error bars indicate standard errors. Significance was determined using *t*-tests. *, *p* < 0.05; **, *p* < 0.01; ***, *p* < 0.001. Uni, un-injection; Ctr, control, injection with ddH_2_O; HD5, injection with HD5.

Parasites are killed by the innate immune system in mosquitoes [28]. Four major signaling pathways have been demonstrated to defend against pathogens, among which the Toll and Imd signaling pathways are considered to be mainly involved in anti-*Plasmodium* defense in mosquitoes [5,29]. To explore whether HD5 treatment activates the Toll or Imd signaling pathways and further influence oocyst development, the key effector molecules in the signaling pathways were evaluated using qRT-PCR. We detected the expression of MyD88, Rel1, and Cactus of the Toll signaling pathway. The expression of both MyD88 and Rel1 was upregulated at 24 hpi and 72 hpi, following HD5 treatment at 12 hpi (Figure 4a,b). The expression of the negative regulatory factor, Cactus, was downregulated at 24 hpi (Figure 4c). This indicates that the Toll signaling pathway was activated. In addition, we detected the expression of Imd and Rel2, which are two key molecules in the Imd signaling pathway. However, there was no significant change in the expression of these genes between the HD5 and control groups (Figure 4d,e), indicating that the Imd signaling pathway was not activated by HD5. These results suggest that the Toll signaling pathway may be activated by HD5 treatment, resulting in the upregulation of TEP1 expression which then results in a decline in oocyst count.

### 3.5. Inhibition of Oocyst Development in An. stephensi by HD5 Could Be Reversed by Toll Signaling Pathway Interference

**Figure 5:**
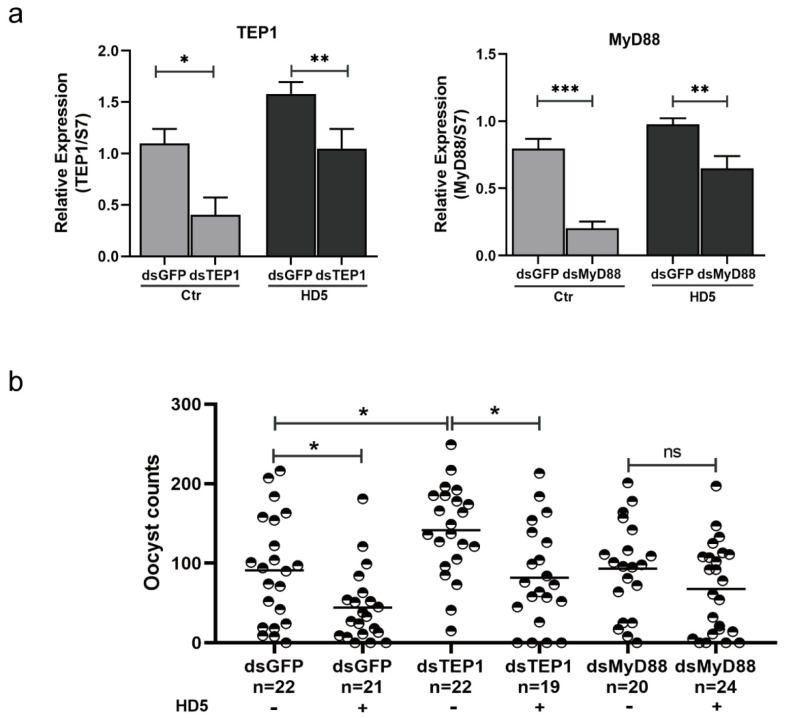
RNAi of TEP1 and MyD88 altered *P. yoelii* oocyst density in HD5-treated and untreated mosquitoes. (**a**) Silencing efficiency of TEP1 and MyD88 expression in mosquitoes by RNAi method. (**b**) Oocyst counts when TEP1 and MyD88 were silenced by RNAi or not in HD5-treated and untreated mosquitoes. Each dot represents oocyst of individual mosquito. Horizontal black bars indicate median values. Significance was determined using *t*-test in (**a**) and by Mann–Whitney tests in (**b**). *, *p* < 0.05; **, *p* < 0.01; ***, *p* < 0.001; ns, no significant difference. Ctr, control, injection with ddH_2_O; HD5, injection with HD5.

To confirm the role of Toll signaling pathway upregulation by HD5 treatment in the inhibition of malaria parasite development in mosquitoes, the gene expression of MyD88 and TEP1 was silenced using RNA interference (RNAi), followed by the observation of oocyst development. The gene expression of TEP1 and MyD88 was efficiently silenced, according to the qRT-PCR results (Figure 5a). As shown in Figure 5b, the inhibitory effect of HD5 on oocyst development decreased slightly when MyD88 expression was silenced by RNAi, indicating that the Toll signaling pathway plays a role in the HD5-mediated transmission blocking of malaria. There were more oocysts in the dsTEP1 group than in the dsGFP group without HD5 treatment, which indicated the role of TEP1 in the killing of *Plasmodium*. In addition, there were fewer oocysts in the HD5 group than in the control group after the injection of dsGFP, which further confirmed the inhibitory effect of HD5 treatment on oocyst development. Nonetheless, HD5 reduced oocyst counts when TEP1 expression was silenced by RNAi, indicating that HD5 treatment may also affect other immune effectors, in addition to TEP1, in the Toll signaling pathway to inhibit oocyst development. In summary, these results suggest that the activation of the Toll signaling pathway by HD5 reduces the susceptibility of *Anopheles* mosquitoes to *Plasmodium*.

## 4. Discussion

New strategies are urgently needed to block malaria transmission. It is worth investigating whether HD5 has a parasiticidal effect on *Plasmodium* and influence on the innate immunity of mosquitoes. We found that the number of oocysts was substantially reduced by the microinjection of HD5 into *Anopheles* mosquitoes, indicating that HD5 can reduce the susceptibility of *Anopheles* mosquitoes to *Plasmodium*. Oocyst counts varied when HD5 was injected intrathoracically into *An. stephensi* at different time points before and after *Plasmodium* infection, which may be related to the development of *Plasmodium* in mosquitoes. Female mosquitoes were infected by ingesting a blood meal containing *Plasmodium* gametocytes. Oocyst development was substantially inhibited when HD5 was administered prior to infection or injected at 6, 12, or 24 hpi. This suggests that HD5 mainly acts on the early stage of sporogony. Each oocyst contains thousands of haploid sporozoites, which then migrate to the salivary glands through the mosquito hemolymph. Thus, the status of oocysts determine following the development of sporozoites and can further affect malaria transmission. However, without an effect on infection rates, HD5 treatment reduced oocysts in mosquitoes, which may result in fewer sporozoites and decrease the vector competence of *Anopheles*. However, there is a limitation of this study, such as the possibility that the rodent malaria parasite may differentiate with human *Plasmodium*. It is necessary to investigate whether HD5 has the same effect on human *Plasmodium* species such as *P. falciparum* in the future.

Our study found that the expression of TEP1 was upregulated by HD5 treatment, indicating that TEP1 is probably involved in the effect of HD5 on the restriction of *Plasmodium*. The silencing of TEP1 expression via RNAi increased the oocyst count, which was consistent with previous reports [30]. However, it did not reverse the effect of HD5 on *Plasmodium* development, indicating the involvement of other effector molecules or the insufficient silencing of TEP1 expression by dsRNA. In *Drosophila*, the inactivation of the Toll and Imd pathways has been used to turn off many immune genes, including antimicrobial peptide genes [31]. The detection and comparison of the expression of key molecules in these two signaling pathways showed that the Toll signaling pathway was upregulated by HD5 injection, whereas the Imd pathway was not affected. The role of the Toll signaling pathway in the inhibitory effect of HD5 on parasite development in *Anopheles* was further confirmed by silencing MyD88, a key upstream molecule in the Toll pathway. This is consistent with the finding that the activation of the Toll signaling pathway in *Anopheles gambiae* by silencing Cactus promotes the activation of the complement-like system in mosquitoes, leading to the elimination of the oocyte cell malaria parasite [32].

Additionally, both the inhibitory effect on malaria parasites and effect on the immune responses of mosquitoes are not HD5-specific. Actually, various physical, chemical, and biological factors have been shown to influence the immune response of mosquitoes. In the present study, ddH_2_O was used as the negative control to maintain the consistency of HD5 solvent and exclude the effect of injection with ddH_2_O. The results of this study using ddH_2_O as the control can reflect the effect of HD5 on the development of malaria parasites in mosquitoes, as well as on the expression of the key molecules of innate immune signaling pathways such as TEP1 and Toll, etc. It does not rule out the possibility that other adventitious factors such as a random sequence polypeptide have the same effect. It is necessary to use a polypeptide without an effect on the immune response of mosquitoes as a negative control to further explore the effect-related sequence characteristics of HD5 in the future.

Many cys-rich peptides, such as phormicins, royalisin, tenecin-1, heliomicin, spodoptericin, gallerimycin, coprisin, and lucifensin, are classified as the insect defensin family [33,34]. They are widely distributed in Diptera, Hemiptera, Hymenoptera, Coleoptera, and Lepidoptera [35], even in the ancient insect order of Odonata [36]. However, it has not been reported that mosquitoes have the ortholog of HD5 with similar inhibitory effect. Researchers have identified various defensin gene isoforms of insects including mosquitoes, which have been sequenced and used as molecular markers for phylogenetic analysis [37]. According to the multiple alignment of human and mosquito defensins and the constructed phylogeny tree, HD5 was most closely related to DEF4 in *An. stephensi* with only 21% homology (Figure S1). So, it is hard to regard DEF4 as the ortholog of HD5 in *An. stephensi*. The differences in the sequences of nucleotides and amino acids between HD5 and mosquito defensins may cause various antimalarial effects. If the ortholog of HD5 in mosquitoes can be found in the future, malaria transmission can be blocked via upregulating its expression. It is also possible to make genetic modifications to mosquito defensin to improve the antimalarial effect in the future.

In addition, a type 1 recombinant defensin (rDef1.3) produced from *Triatoma pallidipennis* showed in vitro lytic activity on *Trypanosoma* and *Leishmania* parasites. The *Leishmania major* parasite was the most affected with 50% of dead cells or with damaged membranes [38]. The silencing of *Phlebotomus papatasi* defensin (PpDef) expression using the RNAi method caused increased parasite levels in the sand fly, indicating that defensin can resist *Leishmania major* infection [39]. The expression of AMPs in mosquitoes is a new approach for killing parasites. The defensin A (DefA) gene has been engineered in *Aedes aegypti* to construct genetically stable transgenic mosquitoes. The expression of DefA can be activated by blood meal and shows antibacterial activity [40]. The expression of cecropin A in transgenic *Anopheles gambiae* can reduce the number of *P. berghei* oocysts [41]. However, overexpressing a single AMP in transgenic mosquitoes is not efficient to interrupt pathogen transmission. The coexpression of cecropin A and defensin A in transgenic *Aedes aegypti* mosquitoes results in a cooperative antibacterial and anti-Plasmodium action. The number of *Plasmodium gallinaceum* oocysts was dramatically reduced in midguts, and no sporozoites were found in the salivary glands of the transgenic mosquitoes [42]. As an effector molecule, human defensin 130 (DEFB130) played a possible role in parasite elimination in macrophages [43]. The coexpression of two or more AMPs in transgenic mosquitoes cooperatively affects *Plasmodium* development. Therefore, HD5 and insect defensins can be engineered in mosquitoes to reduce parasite infection and interrupt parasite transmission. It will provide the possibility of using the cooperation of defensins to enhance the activity of *Anopheles* to defend against *Plasmodium*.

## 5. Conclusions

In conclusion, the present study focused on the inhibitory effect of HD5 on *Plasmodium* development in mosquitoes and its innate immunity-related mechanisms. The results indicated that the intrathoracic injection of HD5 into *An. stephensi* decreased *P. yoelii* infection density by upregulating the Toll signaling pathway. This study can help us not only understand the effect and mechanism of HD5 on the susceptibility of *Anopheles* mosquitoes to *Plasmodium* but also develop new strategies to block malarial transmission.

## Figures and Tables

**Table 1:** Gene primers for quantitative PCR.

Target Gene	Primer Sequence (5′ to 3′)
As-TEP 1	F: ACCGATTGTCCAAGTTCTCG
	R: AGCGCATCTGGTTCTGGTAG
As-MyD88	F: TCGGCGGACAGTGACATTATTACG
	R: TCACGATCCTTCAGACACAGTTGC
As-Rel 1	F: GAACTGGATTCGGTCACGCTAAGG
	R: CGGCAGATAATCAGGTCGGACATG
As-Cactus	F: CGCTTGCAGATGCTAGTGGTCAG
	R: CCGCTGTTCGCTGGCTGTTC
As-Imd	F: CGACCGGAATGCAGGTGTATCAG
	R: CCGCAGAGCCACTCGTTGAAG
As-Rel 2	F: AATTACCCGCATTCTGATCG
	R: CTCCAGCACGTAGTTCACGA
As-S7	F: CTAACGACACGAAGACCACAAGA
	R: CAACCTGCAACGACAGCAAAA

**Table 2:** Specific gene primers for RNAi.

Target Gene	Primer Sequence (5′ to 3′)
T7-dsGFP	F: TAATACGACTCACTATAGGGAGTCAAGTTCAACGTGTCCGGCG
	R: TAATACGACTCACTATAGGGAGAGGACCATTTGATCGCGCTT
T7-dsTEP1	F: TAATACGACTCACTATAGGGAGTCGGGCTGAAGGCGTTGACC
	R: TAATACGACTCACTATAGGGAGTGCCACCTTGAATCGTCTGA
T7-dsMyD88	F: TAATACGACTCACTATAGGGAGAGGTGAGCGTCAAAGAGACG
	R: TAATACGACTCACTATAGGGAGTTTTACTGGCTTGTCCGGCT

## Data Availability

All data generated or analyzed during this study are included in this published article and its Supplementary Materials. The original study data were presented to the authors.

## Supplementary Materials

The following supporting information can be downloaded at https://www.mdpi.com/article/10.3390/tropicalmed9080169/s1, The multiple alignment of the defensins derived from the retrieved sequences from various nucleotide and protein databases is shown in Figure S1.
