# Supplementary material for: Human Defensin 5 Inhibits Plasmodium yoelii Development in Anopheles stephensi by Promoting Innate Immune Response
Source: Trop Med Infect Dis. 2024 Jul 25;9(8):169. doi: 10.3390/tropicalmed9080169 (PMC11360097; doi:10.3390/tropicalmed9080169)
Supplement: Supplementary file 1 [file tropicalmed-09-00169-s001.zip › tropicalmed-3065726-supplementary.pdf]

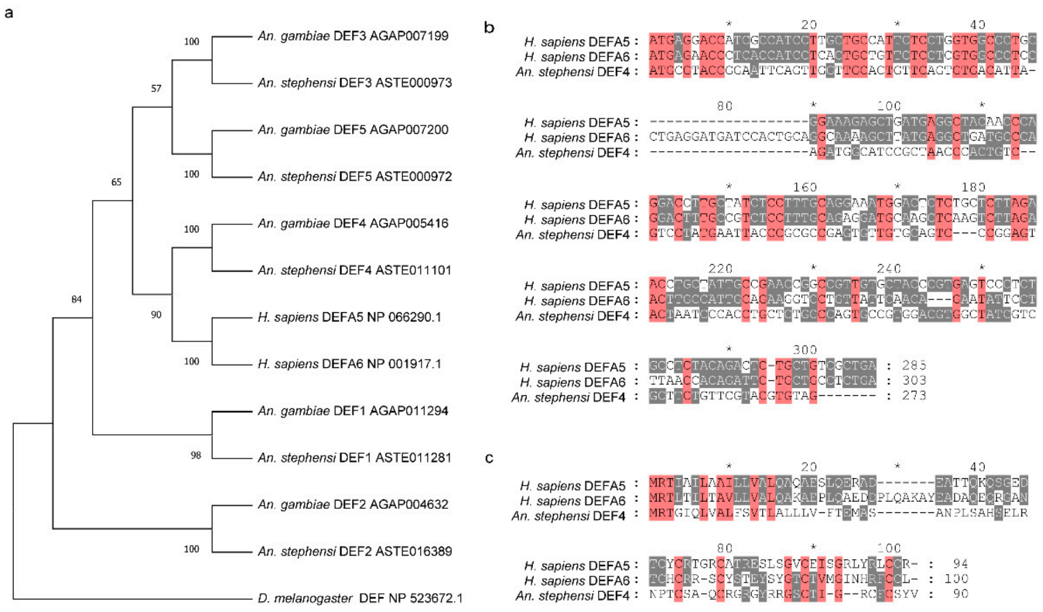

Figure S1. Multiple alignment of the defensins derived from the retrieved sequences from various nucleotide and protein databases. (a) A phylogenetic tree was constructed using the Neighbor-joining method for Defensin amino acid sequences of *Anopheles stephensi* using the DNASTAR and MEGA 11 program. Multiple alignment of nucleotide sequences(b) and amino acid sequences (c) were carried out using CLUSTAL W of BioEdit Version 7.2.5.
